# SUMOylation of EHD3 Modulates Tubulation of the Endocytic Recycling Compartment

**DOI:** 10.1371/journal.pone.0134053

**Published:** 2015-07-30

**Authors:** Or Cabasso, Olga Pekar, Mia Horowitz

**Affiliations:** Department of Cell Research and Immunology, Tel Aviv University, Ramat Aviv, Israel; National Institute of Biological Sciences, Beijing, CHINA

## Abstract

Endocytosis defines the entry of molecules or macromolecules through the plasma membrane as well as membrane trafficking in the cell. It depends on a large number of proteins that undergo protein-protein and protein-phospholipid interactions. EH Domain containing (EHDs) proteins formulate a family, whose members participate in different stages of endocytosis. Of the four mammalian EHDs (EHD1-EHD4) EHD1 and EHD3 control traffic to the endocytic recycling compartment (ERC) and from the ERC to the plasma membrane, while EHD2 modulates internalization. Recently, we have shown that EHD2 undergoes SUMOylation, which facilitates its exit from the nucleus, where it serves as a co-repressor. In the present study, we tested whether EHD3 undergoes SUMOylation and what is its role in endocytic recycling. We show, both *in-vitro* and in cell culture, that EHD3 undergoes SUMOylation. Localization of EHD3 to the tubular structures of the ERC depends on its SUMOylation on lysines 315 and 511. Absence of SUMOylation of EHD3 has no effect on its dimerization, an important factor in membrane localization of EHD3, but has a dominant negative effect on its appearance in tubular ERC structures. Non-SUMOylated EHD3 delays transferrin recycling from the ERC to the cell surface. Our findings indicate that SUMOylation of EHD3 is involved in tubulation of the ERC membranes, which is important for efficient recycling.

## Introduction

Endocytosis controls cell surface associated processes including uptake of molecules, receptor signaling as well as responses to channel activation and transporter activity [1–3]. Using several endocytic mechanisms, the cell sorts internalized cargo toward target sites, through the endosomal system or recycle them back to the plasma membrane [4]. The endocytic pathway involves a large number of proteins, which undergo protein-protein interactions mediated by specific domains [5, 6]. One such module is the Eps15 homology (EH) domain, which mediates interactions with proteins containing a three peptides motif, mostly Asp-Pro-Phe (NPF) [7, 8]. More than 50 eukaryotic proteins were identified as containing at least one EH domain [9, 10], among which is an evolutionarily conserved family, designated EH domain containing (EHDs) proteins [11, 12]. In mammalian cells there are four members, EHD1-EHD4, which share at least 70% sequence identity [11, 13]. In *C*. *elegans* and *Drosophila melanogaster* there is one ortholog, *rme-1* and *Past1*, respectively [14, 15].

The crystal structure of EHD2 as a representative model of EHDs [16] revealed that EHDs appear as dimers, dimerization of which is mediated by a highly conserved, mostly hydrophobic, interface in their G-domain. Dimerization of EHDs enables their interaction with lipids and oligomerization along membranes in a ring like structures [16–18]. Oligomerization of EHD2 around liposomes mediated their tubulation *in-vitro* [16]. However, in a semi-permeabilized cell system, EHD3 was the only family member that mediated membrane tubulation [19]. Tubular association of EHD3 [20] is highly important for its role in controlling trafficking from the early endosomes (EE) to the ERC [21] and recycling from the ERC to the plasma membrane [20, 22, 23]. Its closest homolog, EHD1, has also been demonstrated to control recycling from the ERC to the plasma membrane of proteins internalized via clathrin-dependent [14, 24] and clathrin-independent routes [25]. Interestingly, results from a very recent study showed that ciliary vesicle formation requires EHD1-modulated membrane tubulation [26].

Unlike EHD1 and EHD3, EHD2 regulates internalization [27, 28] by modulating Rac1 activity [28], which controls actin polymerization [29]. In a recent study, we found that EHD2 has a dual cellular role and can also serve as a co-repressor of transcription. Entry of EHD2 into the nucleus depends on a nuclear localization sequence (NLS) present in its helical domain. We also showed that its exit from the nucleus depends mainly on its SUMOylation (SUMO-small ubiquitin like modifier) [30].

SUMO is a small molecule (~11 kDa), resembling ubiquitin in its three-dimensional structure [31, 32]. It covalently attaches to target proteins [33] through the acceptor site, ψKxE (in which ψ is an aliphatic branched amino acid and x is any amino acid) [34, 35]. The enzymatic cycle of SUMOylation is similar to the ubiquitylation cycle [31, 36]. All SUMO proteins are expressed in an immature pro-form, in which they contain a C-terminal stretch of variable length (2–11 amino acids) after an invariant Gly-Gly motif that marks the C terminus of the mature protein [37]. Removal of this C-terminal extension by SUMO-specific proteases and exposing the Gly-Gly motif is a prerequisite for the conjugation of SUMO to its targets [36–38].

A wide range of proteins has been documented to undergo SUMOylation, which affects their stability, localization or activity [39, 40]. At the molecular level, this posttranslational modification changes the surface of a target protein, enabling/disabling interactions with other proteins [32]. Although a number of endocytic proteins have been shown to undergo SUMOylation, EHD2 is the only EHD member shown to be modified by SUMOylation [30].

In the present study we show that EHD3 undergoes Lys^315^ and Lys^511^ SUMOylation. We also show that SUMOylation of EHD3 is important for its localization to the tubular structures of the ERC. Non-SUMOylated EHD3 is concentrated in the perinuclear area of the ERC and delays transferrin recycling, strongly implicating that SUMOylation of EHD3 is important for tubulation of the ERC and efficient recycling.

## Materials and Methods

### Tissue culture cells

HEK293T (human epithelial embryonic kidney cells transformed with SV40) (ATCC no. CRL-3216) and COS-7 (CRL-1651) cells were grown in Dulbecco's Modified Eagle's Medium (DMEM) (Gibco BRL, CA, USA), supplemented with 10% FCS (Beit-Haemek, Israel). All cells were grown at 37°C in the presence of 5% CO2.

### Antibodies

Primary antibodies used were as follows: Mouse monoclonal anti-myc antibody [1:1000 for western blotting (WB), 1:600 for immunoprecipitation (IP), 1:200 for immunofluorescence (IF), Cell Signaling Technology, Inc. Denver, MA, USA, #2276]; Polyclonal rabbit anti-GFP antibodies (1:1000 for WB, Santa Cruz Biotechnology, Dallas, TX, USA, #sc-8334); Polyclonal rabbit anti-Rab11 (1:30 for IF, Invitrogen, Camarillo, CA, USA, #71–5300); Monoclonal mouse anti-EEA1 antibody (1:30 for IF, BD Biosciences, San-Jose, CA, USA, #610456); Monoclonal Mouse anti-HA antibody (1:1000 for WB, Santa Cruz Biotechnology, Denver, TX, USA, #sc-805).

Secondary antibodies included: Peroxide-conjugated goat anti-mouse (1:5000 for WB, #115-035-003); Peroxide-conjugated goat anti-rabbit (1:10,000 for WB, #111-035-144); Cy3-conjugated goat anti-mouse (1:200 for IF, #115-166-072); Rhodamine Red-conjugated goat anti-rabbit **(**1:200 for IF, #111-295-144). All secondary antibodies were from Jackson Immunoresearch Laboratories, West Grove, PA, USA).

### Plasmids

HA–SUMO1 was a gift from Prof. Michael Nevels (Institute of Medical Microbiology and Hygiene, University of Regensburg, Regensburg, Germany).

pEGFP–mEHD3, pcDNA3-myc–hEHD3, pET-EHD3 (and its mutant forms) and pEGFP-EHD1 were already described elsewhere [11, 20, 41]. To create mutant forms of EHD3 [pcDNA3-myc-hEHD3K315R, pcDNA3-myc-hEHD3K511R, pcDNA3-myc-hEHD3K(315+511)R, pcDNA3-myc-hEHD3K315A, pcDNA3-myc-hEHD3K511A, pcDNA3-myc-hEHD3K(315+511)A, pEGFP-C3-mEHD3K315R, pEGFP-C3-mEHD3K511R, pEGFP-C3-mEHD3K(315+511)R, pEGFP-C3-mEHD3K315A, pEGFP-C3-mEHD3K511A, pEGFP-C3-mEHD3K(315+511)A,] *in-vitro* site-directed mutagenesis was performed on pEGFP–mEHD3 and pcDNA3-myc–hEHD3, using the QuikChange site-directed mutagenesis kit (Stratagene Life Technologies, Grand Island, NY, USA), according to the manufacturer’s instructions using primers shown in Table 1.

**Table 1:** Primers used in the present work for in vitro mutagenesis.

CONSTRUCTED PLASMID	NAME OF PRIMER	PRIMER USED
pcDNA3-mychEHD3K315R	hEHD3K315R-F	5’CATCATCAGCTCTCTGAGGAAGGAGATGCCCTCGG3’
	hEHD3K315R-R	5'CCACCACATCAAAGTCAGGCTGGAGGGGCA3'
pcDNA3-myc–hEHD3K511R	hEHD3K511R-F	5'CCACCACATCAAAGTCAGGCTGGAGGGGCA3'
	hEHD3K511R-R	5’ GTGCCCCTCCAGCCTGACTTTGATGAGGTGG3'
pcDNA3-myc–hEHD3K(315+511)R	hEHD3K315R-F	5’CATCATCAGCTCTCTGAGGAAGGAGATGCCCTCGG3’
	hEHD3K315R-R	5'CCACCACATCAAAGTCAGGCTGGAGGGGCA3'
	hEHD3K511R-F	5'CCACCACATCAAAGTCAGGCTGGAGGGGCA3'
	hEHD3K511R-R	5’ GTGCCCCTCCAGCCTGACTTTGATGAGGTGG3'
pEGFP–mEHD3K315R	m.HD3K315R-F	5'CATCATCAGCTCCTTGAGGAAGGAGATGCCCTCAG3'
	mEHD3K315R-R	5'CTGAGGGCATCTCCTTCCTCAAGGAGCTG ATGATG3'
pEGFP–mEHD3K511R	mEHD3K511R-F	5'CCACCTTATCAAAGTCAGGCTAGAGGGGC GAGC AT3'
	mEHD3K511R-R	5'GCTCATGCCCCTCTAGCCTGACTTTGATA AGGTGG3'
pEGFP–mEHD3K(315+511)R	m.HD3K315R-F	5'CATCATCAGCTCCTTGAGGAAGGAGATGCCCTCAG3'
	mEHD3K315R-R	5'CTGAGGGCATCTCCTTCCTCAAGGAGCTG ATGATG3'
	mEHD3K511R-F	5'CCACCTTATCAAAGTCAGGCTAGAGGGGC GAGC AT3'
	mEHD3K511R-R	5'GCTCATGCCCCTCTAGCCTGACTTTGATA AGGTGG3'
pcDNA3-myc–hEHD3K315A	hEHD3K315R-F	5'CATCATCAGCTCTCTGGCGAAGGAGATGCCCTCGG3'
	hEHD3K315R-R	5'CCGAGGGCATCTCCTTCGCCAGAGAGCTGATGATG3'
pcDNA3-myc–hEHD3K511A	hEHD3K511R-F	5'CCACCTTATCAAAGTCAGGCTAGAGGGGC GAGC AT3'
	hEHD3K511R-R	5'GCTCATGCCCCTCTAGCCTGACTTTGATA AGGTGG3'
pcDNA3-myc–hEHDK(315+511)A	hEHD3K315R-F	5'CATCATCAGCTCTCTGGCGAAGGAGATGCCCTCGG3'
	hEHD3K315R-R	5'CCGAGGGCATCTCCTTCGCCAGAGAGCTGATGATG3'
	hEHD3K511R-F	5'CCACCTTATCAAAGTCAGGCTAGAGGGGC GAGC AT3'
	hEHD3K511R-R	5'GCTCATGCCCCTCTAGCCTGACTTTGATA AGGTGG3'
pEGFP–mEHD3K315A	m.HD3K315R-F	5'CATCATAGCTCCTTGGCGAAGGAGATGCCCTCAG3'
	mEHD3K315R-R	5'CTGAGGGCATCTCCTTCGCCAAGGAGCTGATGATG
pEGFP–mEHD3K511A	mEHD3K511R-F	5'CCACCTCATCAAAGTCGCGCTGGAGGGGCAC3'
	mEHD3K511R-R	5'GTGCCCCTCCAGCGCGACTTTGATGAGGTGG3'
pEGFP–mEHD3K(315+511)A	m.HD3K315R-F	5'CATCATAGCTCCTTGGCGAAGGAGATGCCCTCAG3'
	mEHD3K315R-R	5'CTGAGGGCATCTCCTTCGCCAAGGAGCTGATGATG
	mEHD3K511R-F	5'CCACCTCATCAAAGTCGCGCTGGAGGGGCAC3'
	mEHD3K511R-R	5'GTGCCCCTCCAGCGCGACTTTGATGAGGTGG3'

Accession numbers for the cDNAs used are as follows: hEHD1: NM_001282444.1; hEHD3: MN_014600.2; mEHD3: NM_020578.3.

### RNA preparation

Total cellular RNA was isolated using TRizol Reagent (Life-technologies Co. Carlsbad, CA, USA) according to the manufacturer's instructions.

### Transfections

Transfection of COS cells was performed using FuGene 6 Transfection Reagent (Roche Diagnostics, Penzberg, Germany) according to provided instructions. HEK293T cells were transfected using calcium phosphate solutions as described elsewhere [30].

### SDS/PAGE and western blotting

Cell monolayers were washed three times with ice-cold PBS and lysed at 4°C in immunoprecipitaion lysis buffer (10 mM Hepes, pH 8, 100 mM NaCl, 1 mM MgCl2 and 0.5% Nonidet P40) containing protease inhibitors (10 *μ*g/ml aprotinin, 0.1 mM PMSF and 10 *μ*g/ml leupeptin) (Sigma-Aldrich, Rehovot, Israel). Lysates were incubated on ice for 30 min and centrifuged at 10,000g for 15 min at 4°C. Samples containing the same amount of protein were electrophoresed through 10% SDS/PAGE and electroblotted onto a nitrocellulose membrane (Schleicher and Schuell BioScience, Keene, NH, USA). Membranes were blocked with 5% (w/v) non-fat dried skimmed milk powder and 0.1% Tween 20 in TBS (Tris-buffered saline; 20 mM Tris/HCl, 4 mM Tris-base, 140 mM NaCl and 1 mM EDTA) for 30 min at RT and incubated with the primary antibodies overnight at 4°C. The membranes were then washed three times with 0.1% Tween 20 in TBS and incubated with the appropriate secondary antibodies for 1 h at room temperature. After washing, membranes were incubated with enhanced chemiluminescence (ECL) detection reagent (Santa Cruz Biotechnology, Dallas, TX, USA) and analyzed using a luminescent image analyzer (Kodak X-OMAT 2000 Processor, Willoughby, OH, USA or ChemiDoc XRS+, Bio-Rad, Hercules, CA, USA)

### Immunoprecipitation and coimmunoprecipitation

Following 3 washes with ice-cold PBS, cells were lysed on ice in 600µl of lysis buffer (10 mM Hepes, pH 8, 100 mM NaCl, 1 mM MgCl_2_ and 0.5% Nonidet P40) containing protease inhibitors (10 μg/ml aprotinin, 0.1 mM PMSF and 10 μg/ml leupeptin) (Sigma-Aldrich, Rehovot, Israel). The corresponding supernatants were pre-cleared for 2 hours at 4°C with the desired antibody, immobilized on protein A agarose (Roche Dianostics, Penzberg, Germany). Following four washes with lysis buffer containing protease inhibitors as above proteins were eluted for 5 min at 100°C with 5x sample buffer, electrophoresed through 10% SDS-PAGE and blotted. The corresponding blot was interacted with the appropriate antibodies.

### Immunofluorescence and Confocal Microscopy

For immunofluorescence and confocal microscopy, cells were grown on cover glasses (Marienfeld, Lauda-Konigshofen, Germany) and transfected with the desired plasmids. Twenty-four hours later the cells were washed three times with PBS and fixed with 4% paraformaldehyde for 15 minutes at RT, followed by three additional PBS washes. Permeabilization was performed with 0.1% Triton X-100 in 50 mM Tris pH7.2 for 3 minutes, after which cells were washed with PBS and incubated in blocking buffer (20% normal goat serum and 1% BSA in PBS) for 30 minutes at RT. Cells were incubated with the appropriate antibody that was diluted in PBS containing 1% BSA for 1 hour at RT, followed by three PBS washes. The cover glasses were then incubated with the secondary antibody for 1 hour at RT. Following washes with PBS they were mounted using fluorescent mounting medium (E19-15, Golden bridge Life science, WA, USA).

Cells were examined using Zeiss LSM 510 META confocal microscope. For quantitative studies, all images of a given experiment were exposed and processed identically. Captured images were analyzed using ImageJ software. Pixel intensity was used to quantify fluorescence in the indicated experiments. Data was statistically evaluated using Student’s *t* test.

### Protein expression in BL21 Bacteria

An overnight culture of BL21 bacterial, transformed with pET-EHD3 (or its mutant forms), was diluted 1:10 in fresh LB and grown to OD_600_ = 0.8–1. IPTG was added to a final concentration of 0.1mM and expression was allowed to proceed for 2 hours at 37°C. Following a wash with PBS, bacteria were centrifuged and resuspended in native lysis buffer containing 50 mM Na_2_HPO_4_, 300 mM NaCl and 10 mM imidazole. The lysates were incubated with 1 mg/ml lysozyme on ice for 30 minutes, followed by centrifugation for 10 minutes at 15,000g at 4°C. The protein concentration in the supernatant was measured using NanoDrop ND-1000 UV-Vis Spectrophotometer (NanoDrop, DE, USA). Samples containing the same amount of protein were electrophoresed through SDS-PAGE and the corresponding gel was stained with SeeBand (Gene Bio Applications Ltd., Yavne, Israel).

### *In-vitro* SUMOylation

BL21 bacteria, transformed with pET-EHD3 plasmids, were lysed as described above. His-tagged proteins were isolated from the lysates, prepared as described above, using nickel (Ni-NTA) beads (QIAGEN, Hilden, Germany). *In-vitro* SUMOylation assay was performed using *in-vitro* SUMOylation kit (LAE Biotech International, Rockville, MD, USA), according to the manufacturer’s instructions. Briefly, at least 2 μg of purified His-tagged protein was incubated with 0.1 μg of human SUMO1 in the presence of 15 ng of SAEI/SAEII, 0.1 μg of UBC9 and 10XSUMOylation reaction buffer, containing 200 mM Hepes pH 7.5, 50 mM MgCl_2_, and 20 mM ATP (LAE Biotech International, Rockville, MD, USA) in a final volume of 20 μl, for 2 hours at 37°C. The reaction products were subjected to SDS-PAGE and the corresponding blots were interacted with anti His antibody.

### "In-cell" SUMOylation

Forty-eight hours after transfection of HEK293T cells, they were lysed in 200 *μ*l of denaturing buffer (1% SDS, 50 mm Tris/HCl, pH 7.4, and 140 mM NaCl) by boiling for 10 min after vigorous vortex. Renaturation buffer (800 *μ*l; 2%Triton X-100, 50 mM Tris/HCl, pH 7.4, and 140 mM NaCl) was added and, following centrifugation for 15 min at 10000*g* at 4°C, the supernatants were subjected to immunoprecipitation and western blot analysis as described above.

### Transferrin recycling

Cells, grown on cover glasses, were incubated for 30 minutes in binding medium. Following 5 minutes incubation with 10 μg/ml Alexa-546 conjugated transferrin (Molecular probes, Grand Island, NY, USA) at 37°C, cells were washed three times with PBS and incubated with chase medium (DMEM free, 20% dialysed FCS (dFCS), 20mM Hepes pH7.2, 50 μM deferoxamine, Holo transferrin X100). At the end of the chase, cells were rapidly cooled to 4°C, incubated with citrate buffer for 2 minutes, washed three times with cold PBS and fixed with 4% paraformaldehyde (Merck, Darmstadt, Germany). The fixed cells were mounted for microscopy using DAPI containing fluorescent mounting medium (Golden Bridge international Inc. City of Industry, CA, USA).

Captured images were analyzed using ImageJ software. Pixel intensity was used to quantify fluorescence in the indicated experiments. Data was statistically evaluated using Student’s *t* test.

### Quantitative measurement of recycling by Flow Cytometry

Cells were incubated for 30 minutes in binding medium. Following 5 minutes incubation with 10μg/ml Alexa-647 conjugated transferrin (Molecular probes, CA, USA), cells were washed three times with PBS and chased for the indicated times at 37°C. At each time point, cells were washed with PBS, removed from the dish with warm trypsin and transferred to pre-cooled tubes containing 250μl ice-cold DMEM and pelleted by centrifugation. Cell pellets were immediately fixed in 250μl of 4% paraformaldehyde. At least 5000 cells were analyzed.

## Results

### Mutations in the putative SUMOylation sites of EHD3 change its ERC localization

**Fig 1:**
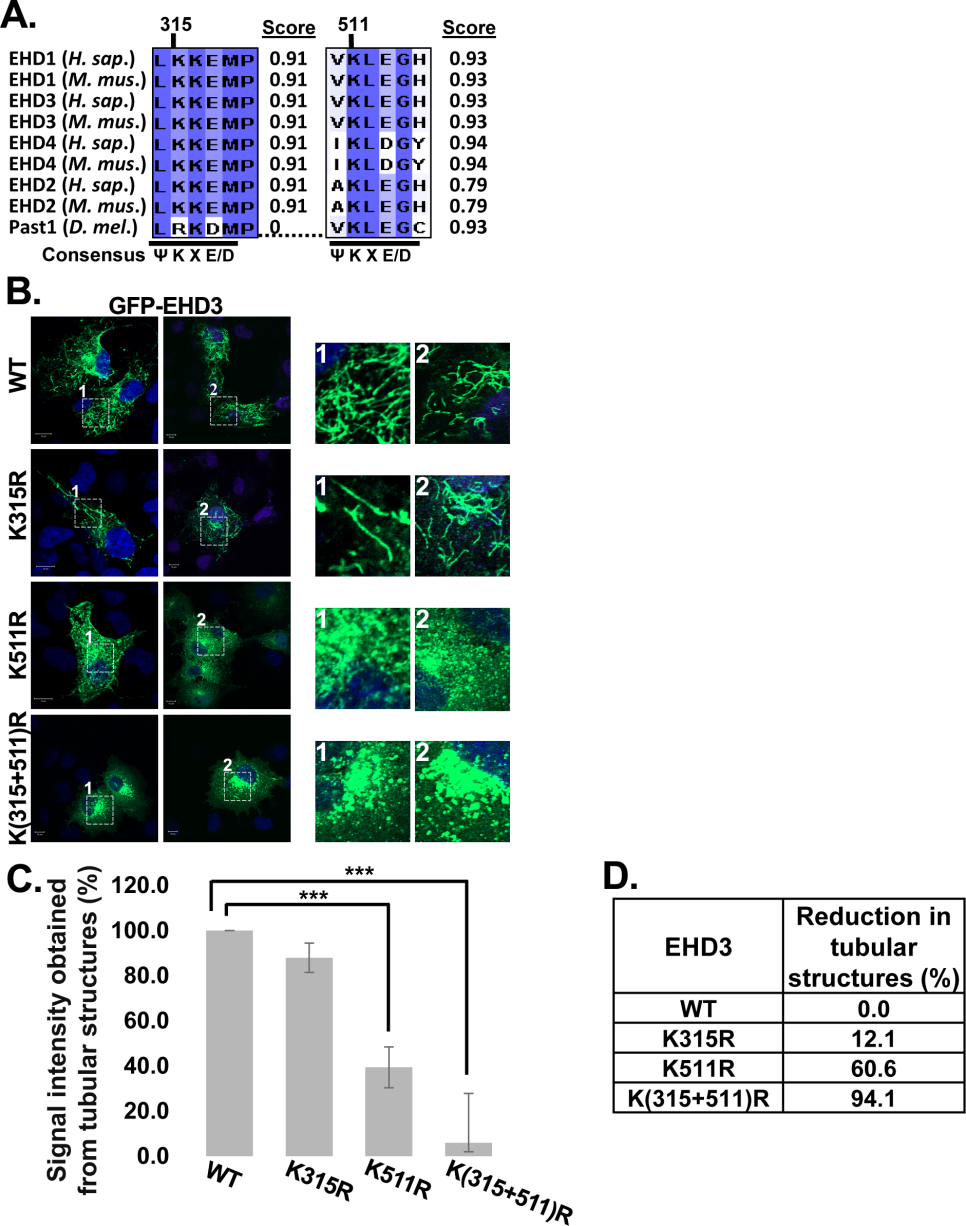
Localization of predicted SUMOylation mutants of EHD3. **A.** Multiple alignment of potential SUMOylation sites in different EHD homologs and their scores (given by SUMOplot™ Analysis Program). The positions of consensus SUMOylation sites are underlined. Position numbers are relevant to human EHD3. **B.** COS-7 cells were transiently transfected with wt GFP-EHD3 or its SUMOylation mutants: GFP-EHD3K315R, GFP-EHD3K511R, GFP-EHD3K(315+511)R. Twenty-four hours later cells were fixed with 4% paraformaldehyde and visualized using confocal microscopy. Right panels depict enlarged regions of the cells and show tubular structures of EHD3. Scale bars represent 10 μm. **C.** Quantification of signal intensity obtained from tubular structures (%) longer than 2 μm in length of either wt or its SUMOylation mutants. The level of signal in the wt sample was considered 100%. ***P<0.0001. Eighty to 100 cells were analyzed for each type of EHD3 variant. **D.** Shown is the percent reduction in tubular structure signal, calculated from the mean values.

All mammalian EHDs have one or two putative SUMOylation sites with scores over 0.90 (Fig 1A). We have shown in the past that SUMOylation of EHD2 is important for its exit from the nucleus [30]. Here, we extended our study to test whether EHD3 undergoes SUMOylation and what function it serves, taking into consideration that EHD3 controls trafficking from the early endosomes to the ERC [21] and from the ERC to the plasma membrane [20, 22]. Thus, we created three variants of EHD3, altered at the predicted SUMOylated Lys^315^ and Lys^511^ (EHD3K315R and EHD3K511R, respectively), and the double mutant [EHD3K(315+511)R] (Fig 1A), and tested their cellular localization in COS transfected cells. As shown in Fig 1B and 1C wt EHD3 and EHD3K315R variants were localized to the tubular structures, with a slight reduction in the amount of GFP-EHD3K315R stained tubules compared to wt EHD3. A significant decrease in the number of EHD3 stained tubular structures was observed for the EHD3K511R variant, while the double mutant [EHD3K(315+511)R] lost its tubular localization and was vesicular. These results suggested that SUMOylation of EHD3 on both Lys^315^ and Lys^511^ is essential for the localization of ehd3 to the tubular structures and that the effect of two sites is synergistic (Fig 1D). Similar results were observed in COS cells, transfected with the different EHD3 SUMOylated variants, in which the predicted SUMOylated lysines were mutated to alanines (S1 Fig). These findings confirmed that EHD3 SUMOylation on Lys^315^ and Lys^511^ is important for its tubular localization and that this effect is synergistic. The results also confirmed that the change of either lysine to alanine (ie: charge change) or lysine to arginine (ie: no change in charge) has the same physiological effect, similarly to what we have shown for EHD2 SUMOylation[30].

**Fig 2:**
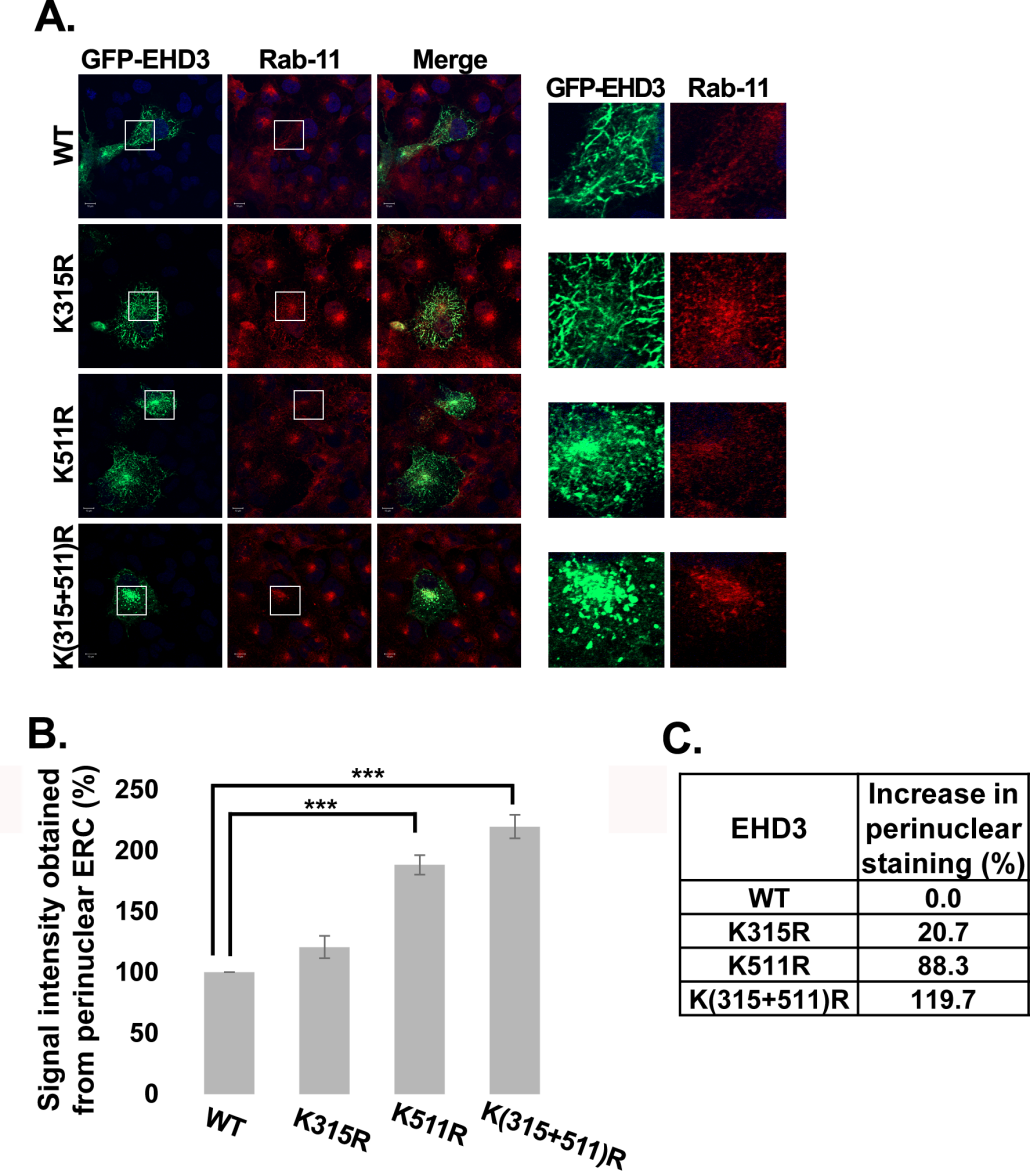
SUMOylation affects ERC localization of EHD3 **A.** COS-7 cells were transiently transfected with plasmids expressing GFP-EHD3 or its SUMOylation mutants. Twenty-four hours later cells were fixed with 4% paraformaldehyde, permeabilized and incubated with anti-Rab11 antibody. Detection was performed with rhodamine conjugated goat anti-rabbit antibodies. The results were visualized using a confocal microscopy (left panel). Right panels depict enlarged regions of the cells. Scale bars represent 10 μm. **B.** Quantification of signal intensity obtained from the perinuclear area of the ERC (the signal was measured from non-tubular structures or tubules, which are less than 2 μm in length). About 40 cells were analyzed for each type of protein. The level of signal in the wt sample was considered 100%. **C.** The percent increase in perinuclear signal, calculated from the mean values.

**Fig 3:**
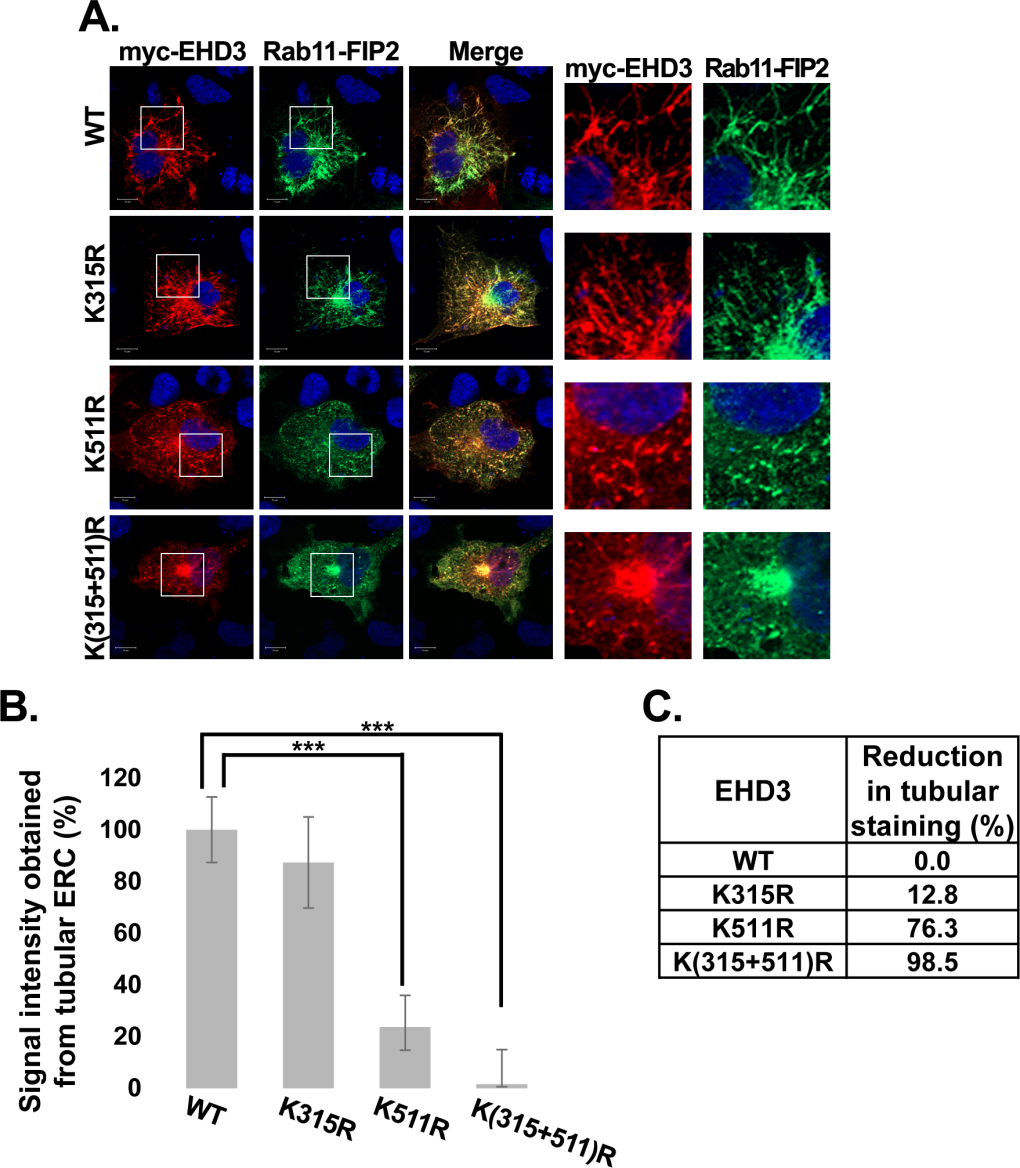
SUMOylation plays an important role in EHD3 localization to recycling endosomal tubules. **A.** COS-7 cells were transiently transfected with plasmids expressing myc-EHD3 or its SUMOylation mutants together with GFP-Rab11-FIP2 (Rab11-FIP2, a kind gift of Prof. Steve Caplan, Nebraska, USA, accession no. NM_014904.2). Twenty-four hours later cells were fixed with 4% paraformaldehyde, permeabilized and incubated with anti-myc antibody. Detection was performed with rhodamine conjugated goat anti-mouse antibodies. The results were visualized using a confocal microscopy (left panel). Right panels depict enlarged regions of the cells. Scale bars represent 10 μm. **B.** Quantification of signal intensity obtained from tubular structures (%) longer than 2 μm in length of either wt EHD3 or its SUMOylation mutants. **C.** Shown is the percent reduction in tubular structure signal, calculated from the mean values.

Since we have shown in the past that EHD3 localizes to endocytic recycling tubules [20], we tested whether the SUMOylation variants of EHD3 localize to Rab11-positive ERC structures [42]. All EHD3 variants colocalized with Rab11 (Fig 2). WT and K315R variants colocalized with Rab11 in typical tubular structures (of size longer than 2 μm), while EHD3K511R colocalized in shorter tubules (less than 2 μm in length). However, the double mutant lost almost completely its tubular localization and concentrated in the perinuclear area of the ERC (defined as closest to the nucleus area, marked by colocalization with Rab11) (Fig 2A). Quantification of perinuclear, non-tubular, ERC staining (Fig 2B and 2C) showed a 2.2 fold increase in the perinuclear signal of the double EHD3 mutant compared to wt EHD3 while EHD3K511R variant presented 1.85 fold increase, indicating the important role of Lys^511^ in the localization of EHD3 to the tubular structures. Although there was only a slight increase in the signal of perinuclear, non-tubular GFP-EHD3K315R compared to wt EHD3, it is likely that Lys^315^ has a minor contribution to the phenotype of the double mutant variant. We also tested the colocalization of EHD3 and its SUMOylation mutants with a known marker for recycling ERC tubules, Rab11-FIP2 [21]. All EHD3 variants colocalized with transfected Rab11-FIP2 (Fig 3). WT and K315R variants colocalized with Rab11-FIP2 in typical tubular structures, while EHD3K511R colocalized with this ERC marker in shorter tubules (less than 2 μm in length). However, the double mutant of EHD3 and transfected Rab11-FIP2 lost almost completely their tubular localization and both concentrated in the perinuclear area of the ERC (Fig 3A). These results are in agreement with published data, which showed that EHD3 modulates localization of Rab11-FIP2 [21]. Quantification of tubular ERC staining (Fig 3B and 3C) revealed almost complete loss of double mutant of EHD3 from the ERC tubular structures. EHDK511R presented 4 fold decrease in the tubular ERC signal, while EHD3K315R showed 1.2 fold decrease, reinforcing our observation that EHD3 SUMOylation on both sites has a synergistic effect. These data imply that SUMOylation of EHD3 is involved in regulation of its localization to the peripheral tubular recycling endosomes and disruption of this posttranslational modification results in accumulation of EHD3 in the perinuclear, non-tubular fraction of the ERC.

### SUMOylation of EHD3

**Fig 4:**
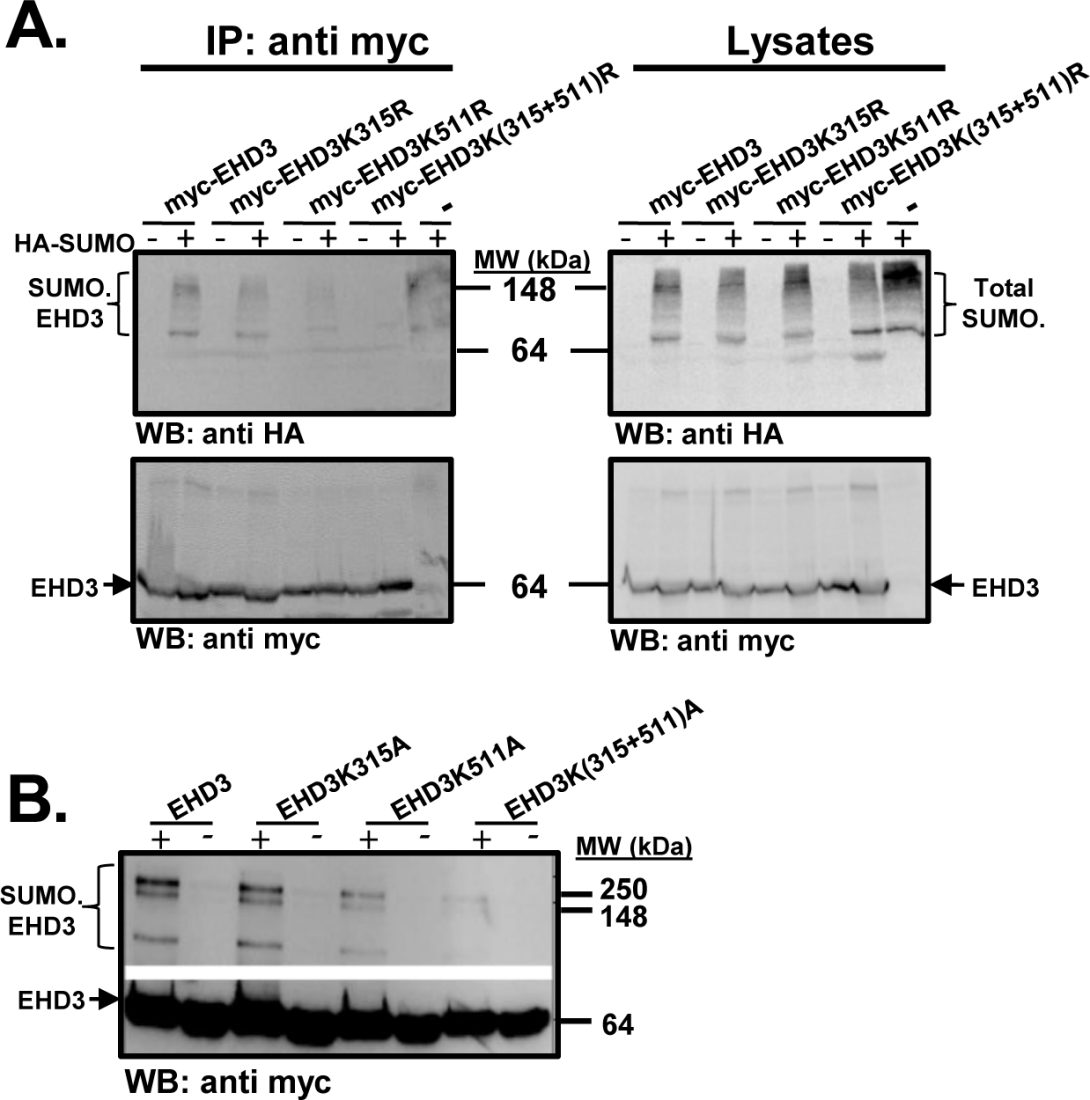
EHD3 undergoes SUMOylation. **A.** Lysates of HEK293T cells, transiently transfected with myc–EHD3 or its SUMOylation mutants [EHD3K315R, EHD3K511R, EHD3K(315R+511)R] and HA–SUMO, were coimmunoprecipitated with anti-myc antibody. The immunoprecipitates were subjected to SDS-PAGE and the corresponding blot was probed with anti-HA (to visualize SUMOylation) and anti-myc (to visualize the EHD3 variant) antibodies. In parallel, 5% of the lysates were subjected to SDS-PAGE and the corresponding blot was probed with anti-HA antibody (in order to assess transfection with SUMO1) and anti-myc antibody (to follow presence of transfected EHD3 variant).**B**. Two micrograms of bacterially purified EHD3 or its SUMOylation mutants (EHD3K315A, EHD3K511A, EHD3K315A/K511A) were incubated with human SUMO1 as detailed in Materials and Methods. The reaction products were subjected to SDS-PAGE and the corresponding blot was incubated with anti-His antibody. IP: immunoprecipitation; WB: western blot.

Aiming at showing that EHD3 undergoes SUMOylation, we tested association of SUMO with EHD3 in cell lysates, prepared from COS cells, transfected with HA-SUMO and different variants of myc-tagged EHD3 expressing plasmids. The results indicated that either wt EHD3 or the EHD3K315R mutant coimmunoprecipitated with HA-SUMO almost at the same level and created a typical ladder of increasing protein masses. However, the Lys^511^ mutant form of EHD3 presented a significant decrease in its association with SUMO in comparison to wt EHD3. Moreover, the EHD3 double mutant showed almost no association with SUMO protein (Fig 4A). To confirm the ability of EHD3 to undergo SUMOylation, *in-vitro* SUMOylation experiments were performed using bacterially expressed proteins. *In-vitro* sumoylation of wt and K315A EHD3 variants resulted in the appearance of several high molecular weight forms of EHD3 (Fig 4B). Mutation in Lys^511^ led to a significant decrease in the appearance of modified forms of EHD3, while double mutation in both Lys^315^ and Lys^511^ resulted in an almost complete disappearance of the SUMOylated forms of EHD3 in comparison to wt EHD3 or the single K315AEHD3 mutant (Fig 4B), confirming SUMOylation of EHD3 on Lys^315^ and Lys^511^.

In summary, our SUMOylation experiments strongly indicated the importance of both Lys^315^ and Lys^511^ for functional SUMOylation of EHD3.

### SUMOylation has a dominant negative effect on EHD3 localization

**Fig 5:**
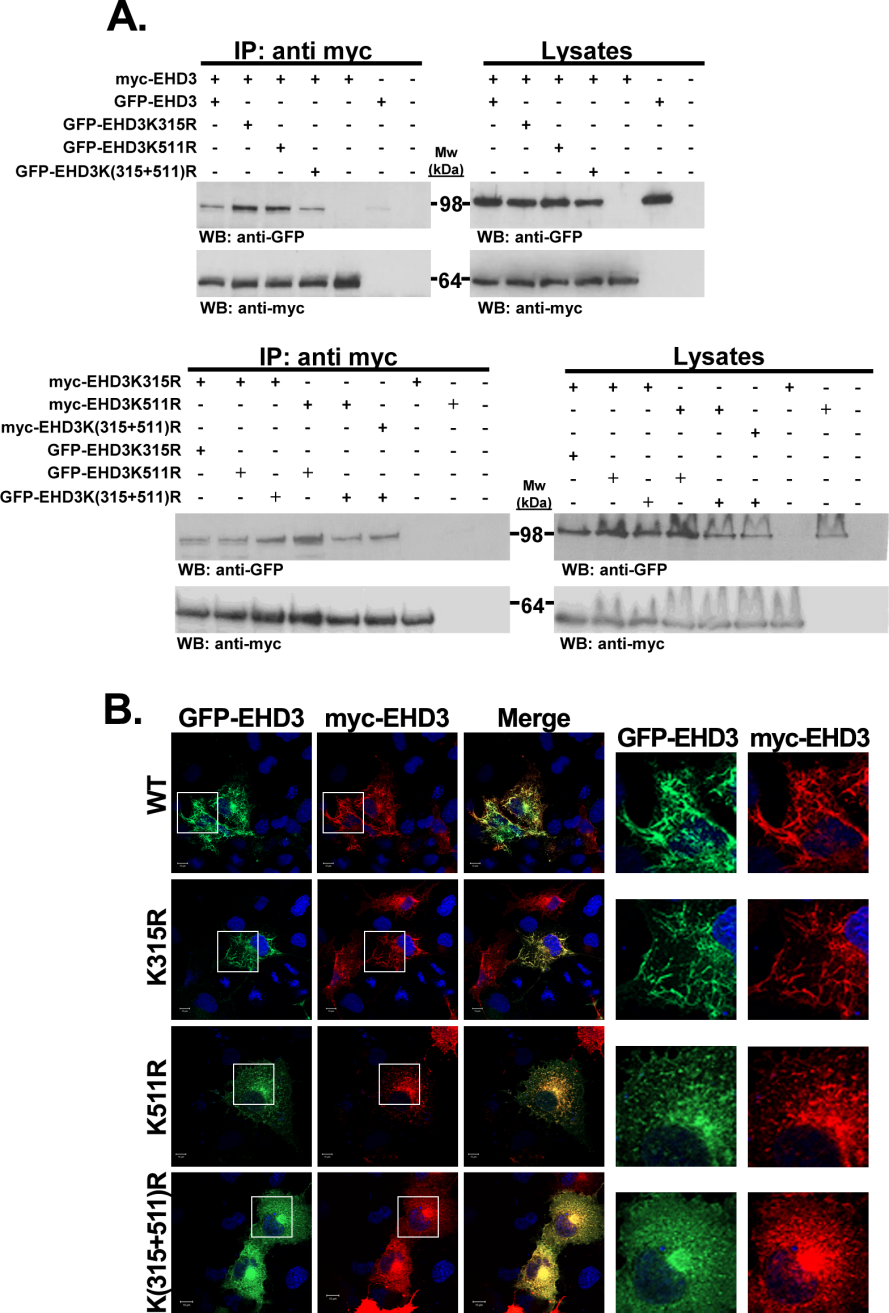
The effect of SUMOylation on dimerization of EHD3. **A**. Lysates of HEK293T cells, transiently transfected with different combinations of EHD3 variants, were coimmunoprecipitated with anti-myc antibody. The immunoprecipitates and 5% of the cell lysates were subjected to SDS-PAGE and the corresponding blots were interacted with anti-myc and anti-GFP antibodies. IP: immunoprecipitation; WB: western blot. **B.** COS-7 cells were transiently cotransfected with plasmids expressing myc-EHD3 together with either GFP-EHD3 or its SUMOylation mutants [EHD3K315R, EHD3K511R, EHD3K(315+511)R]. Twenty-four hours later cells were fixed with 4% paraformaldehyde and visualized (left panel). Right panels depict enlarged regions of the cells. Scale bars represent 10 μm.

EHD2 form dimers, which allow their membrane binding. Further oligomerization of EHD2 allows membrane tubulation (bending) [16]. Membrane tubulation has been shown recently for EHD3 as well [19]. Since we suggest a role for SUMOylation in regulating localization of EHD3 to recycling endosomal tubules, we tested whether it plays a role in dimerization of EHD3. To this aim, coimmunoprecipitation was performed on lysates prepared from HEK293T cells, transfected with plasmids expressing different tagged wt or mutant variants of EHD3. The results depicted in Fig 5A indicated that any variant of EHD3 (wt, single mutant or double mutant) was present in the same complex with either wt or any of the EHD3 SUMOylation mutants. These results strongly suggest that SUMOylation does not affect dimerization. We next tested whether SUMOylation affects localization of EHD3 dimers to the tubular structures. We did so by immunostaining COS cells, transiently transfected with myc-EHD3 and all GFP-EHD3 SUMOylation variants, with the corresponding antibodies. The results strongly indicated that myc-EHD3 colocalized with all GFP-EHD3 SUMOylation variants and were consistent with the immunoprecipitation results (Fig 5B). In cells expressing myc-EHD3 together with either GFP-EHD3 or GFP-EHD3K315R colocalization appeared mainly to the tubular structures. In contrast, myc-EHD3 poorly localized to the tubular structures when it was expressed together with GFP-EHD3K511R and hardly did so in tandem with GFP-EHD3K(315+511)R. Taken together, these results indicate that SUMOylation is not necessary for EHD3 dimerization. However localization of EHD3 (most probably as oligomers) to tubular recycling endosomes depends on SUMOylation of both monomers.

**Fig 6:**
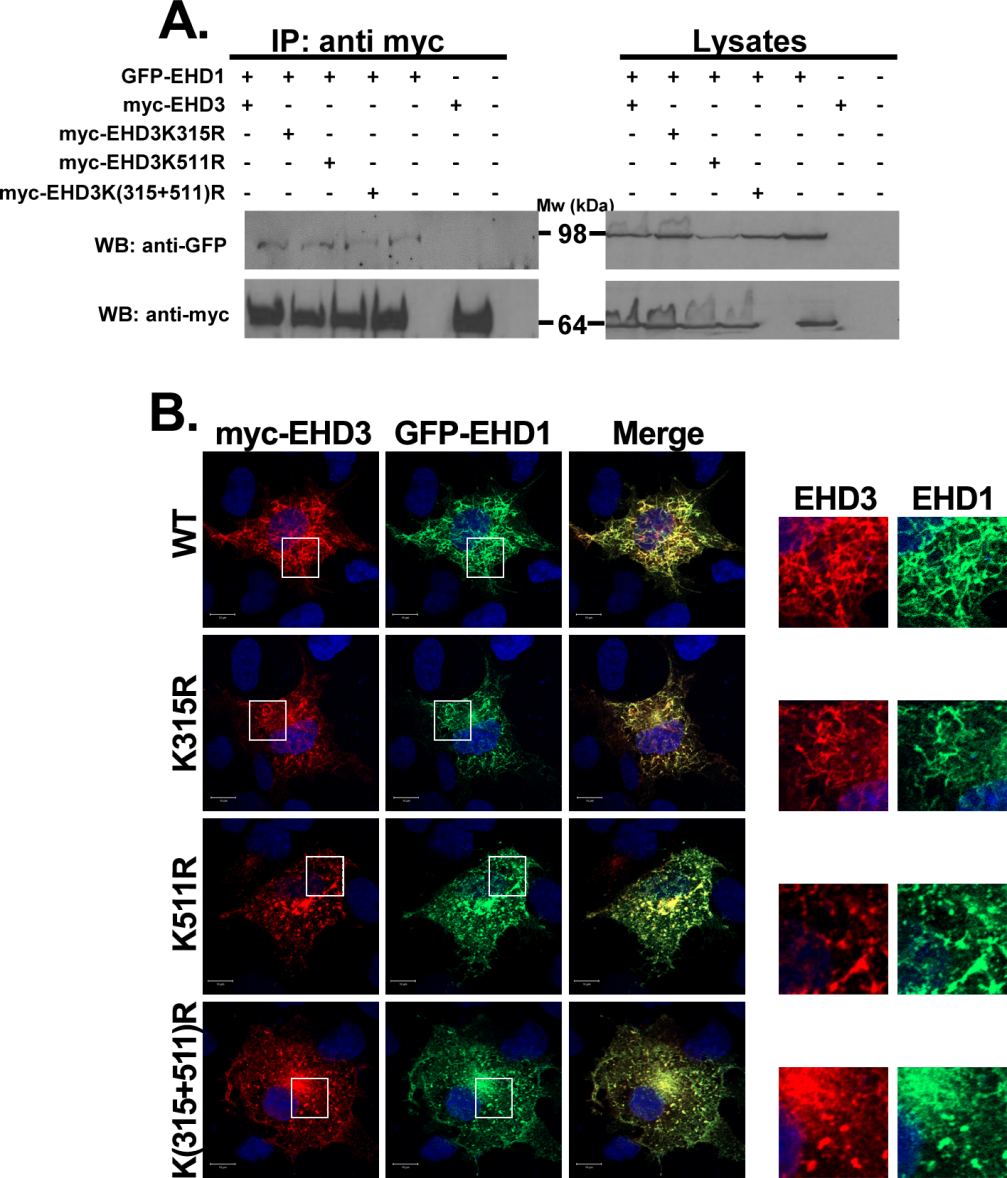
The effect of EHD3 SUMOylation on EHD1 localization. **A**. Lysates of HEK293T cells, transiently cotransfected with GFP-EHD1 and either wt or one of the SUMOylation mutants of EHD3, were coimmunoprecipitated with anti-myc antibody. The immunoprecipitates and 5% of cell lysates were subjected to SDS-PAGE and the corresponding blots were interacted with anti-myc and anti-GFP antibodies. IP: immunoprecipitation; WB: western blot. **B.** COS-7 cells were transiently cotransfected with plasmids expressing GFP-EHD1 together with either myc-EHD3 or its SUMOylation mutants [EHD3K315R, EHD3K511R, EHD3K(315+511)R]. Twenty-four hours later cells were fixed with 4% paraformaldehyde and visualized (left panel). Right panels depict enlarged regions of the cells. Scale bars represent 10 μm.

EHD1, another EHD family member, interacts with EHD3 [20] through their EH-NPF motifs [21]. Also, EHD3 was shown to regulate tubular localization of EHD1 [20]. Therefore, it was interesting to test whether SUMOylation of EHD3 plays a role in this interaction and whether it affects EHD1 localization. Results of coimmunoprecipitation analysis of lysates from HEK293 cells, transfected with plasmids expressing the four variants of EHD3 (Fig 6A), showed that interaction between EHD1 and EHD3 does not depend on EHD3 SUMOylation. However, EHD1 lost its tubular localization in cells expressing EHD3K(315+511)R variant and concentrated in the perinuclear area (Fig 6B). It localized both to very short tubules and vesicular structures in cells expressing EHD3K511R (Fig 6B). However, in cells expressing either EHD3K315R or wt EHD3 variants, EHD1 localized to long tubules. These results imply that SUMOylation of EHD3 is involved in the formation of tubular ERC and therefore, affects both EHD3 and EHD1 localization to the peripheral tubular recycling endosomes".

### SUMOylation of EHD3 affects recycling from the ERC

**Fig 7:**
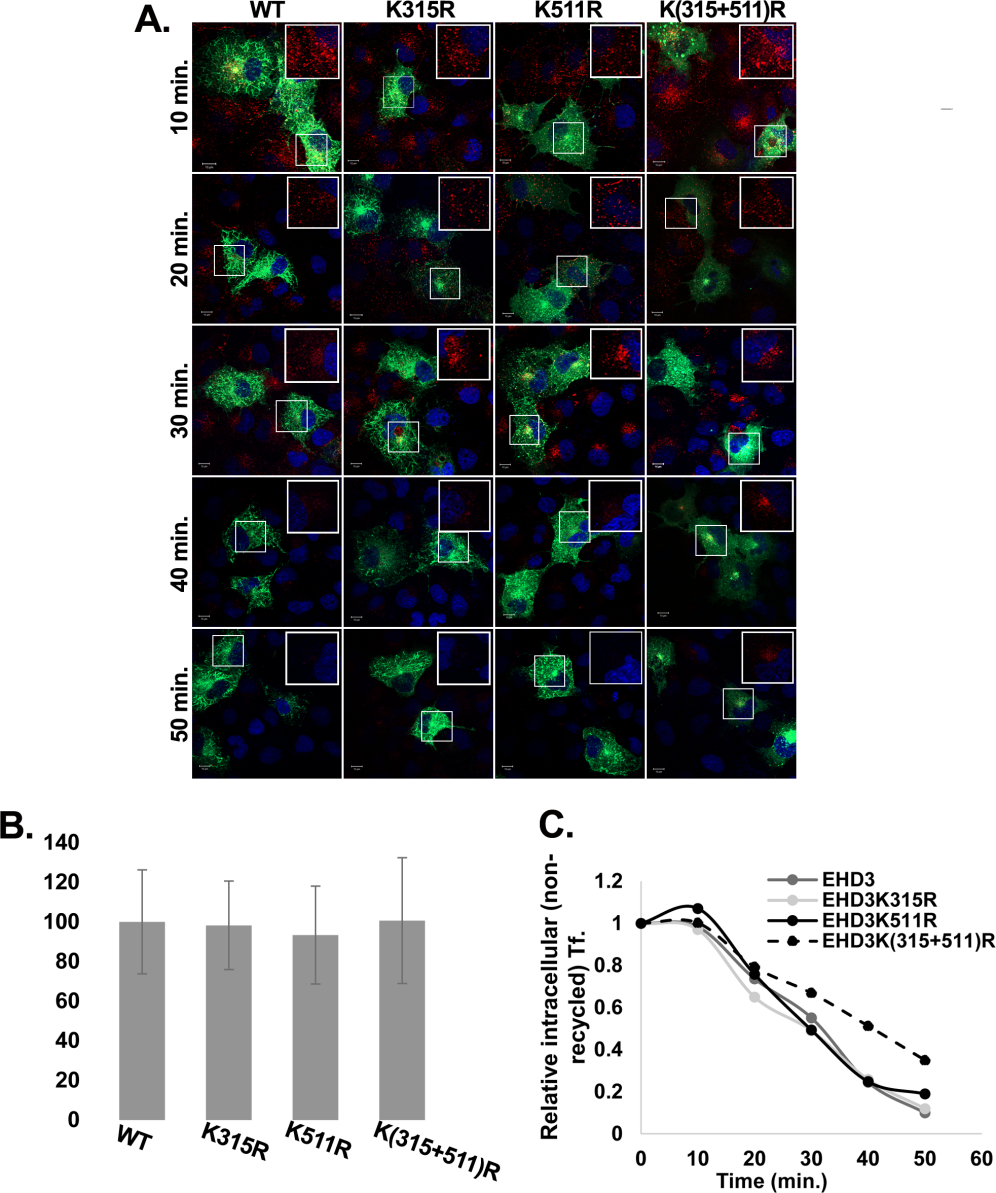
Delay in transferrin recycling in cells expressing SUMOylation mutants of EHD3. **A**. COS-7 cells were transiently transfected with plasmids expressing GFP-EHD3 or its SUMOylation mutants [EHD3K315R, EHD3K511R, EHD3K(315+511)R]. Twenty-four hours later, cells were starved for thirty minutes, incubated with Alexa-546 conjugated transferrin for five minutes at 37°C and chased for the indicated times. Cells were cooled to 4°C, washed with cold citrate buffer for 2 min, fixed with 4% paraformaldehyde and stained with DAPI to visualize the nuclei. Insets depict enlarged regions of transfected cells and show transferrin only. Scale bars represent 10 μm. **B.** Quantification of internalized transferrin was performed by measuring the signal obtained from Alexa-546-conjugated transferrin after 10 min of chase. At least eighty cells from each group were analyzed by ImageJ software. **C.** Quantification was performed by counting the number of cells with intracellular Alexa-546-conjugating transferrin (Tf.). At least 130 cells from three independent assays were counted for each group. The number of positive cells in the wt sample was considered 1.

A recent study showed that knockdown of EHD3 leads to a significant decrease in the tubular structures of the ERC [19]. Such a decrease might serve as a critical factor in recycling of proteins through the ERC. Since inhibition of EHD3 SUMOylation resulted in the absence of EHD3 on tubular structures, it could reflect the need for EHD3 SUMOylation in formation of tubular recycling endosomes. Absence of these endosomes should affect recycling from the ERC to the plasma membrane. To test this possibility, we performed a transferrin-recycling assay. As evident from the results, presented in Fig 7, after ten minutes of chase, the pattern of punctate transferrin (Fig 7A) as well as its intracellular levels (Fig 7B) indicated comparable internalization rate in cells expressing all EHD3 variants. After thirty minutes of chase, most transferrin reached the ERC in cells transfected with all the different EHD3 variants (Fig 7A and 7C). After forty and fifty minutes of chase (Fig 7A and 7C) most labeled transferrin has disappeared from the ERC in cells expressing wt EHD3 or one of its single SUMOylation mutants. However, in cells expressing the double mutant EHD3K(315+511)R, transferrin, concentrating in the perinuclear non-tubular region of the ERC, was detected even after fifty minutes of chase (Fig 7A and 7C). Quantification revealed a twenty percent delay in transferrin recycling in cells that expressed the double mutant in comparison to cells expressing either wt EHD3 or the single SUMOylation mutants (Fig 7C).

**Fig 8:**
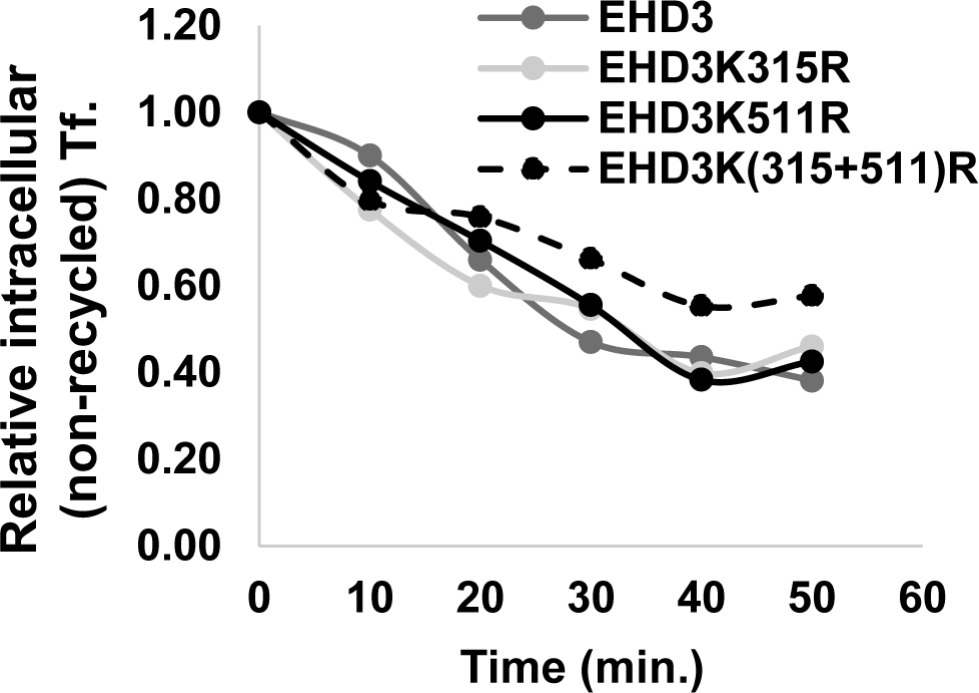
Delay in transferrin recycling in cells expressing SUMOylation mutants of EHD3. COS-7 cells were transiently transfected with either GFP-EHD3 or its SUMOylation mutants. Twenty-four hours later cells were serum starved and incubated with Alexa-647 transferrin for 5 minutes at 37°C. After washing and removing unbound transferrin, the cells were incubated in full media containing excess Holo-transferrin and chased for the times indicated. Cells were harvested by a brief trypsinization, fixed, and analyzed by flow cytometry to determine levels of internal Alexa-647-transferrin (Tf.). At least 7000 cells from two independent assays, were subjected to FACS analysis. The number of positive cells in the wt sample was considered 1.

To confirm the results, the kinetics of transferrin recycling was assayed by flow cytometry (Fig 8). To this end, COS cells, transiently transfected with either GFP-EHD3 or the different SUMOylation mutants, were pulsed with transferrin for five minutes and chased for different times after which they were analyzed by FACS. We noticed a twenty to thirty percent delay in transferrin recycling in cells expressing the EHD3 double mutant [EHD3K(315+511)R] in comparison to cells that expressed wt EHD3 or its single mutants.

The presented results indicate that SUMOylation of EHD3 on both sites plays an important role in controlling the rate of transferrin recycling. Since recycling depends on the presence of endocytic recycling tubules, we assume that EHD3 SUMOylation mediates tubulation of ERC, without which there is a delayed recycling to the plasma membrane.

## Discussion

In the present study, we investigated whether EHD3 undergoes SUMOylation and if so, how this modification affects its function and/or localization. Our results showed that SUMOylation of EHD3 on Lys^315^ and Lys^511^ is an essential modification for its localization to recycling endocytic tubules (Figs 1–3). The experiments were performed using an endogenous ERC marker, Rab11 and a transfected marker, Rab11-FIP2. We found that SUMOylation has a dominant negative effect on tubular localization of EHD3. Moreover, we found that SUMOylation of EHD3 affects also EHD1 localization to the ERC tubules. Non-SUMOylated EHD3 concentrated in a perinuclear area, resulting in a delay in transferrin recycling from the ERC to the plasma membrane. Only in the case of complete ablation of tubular structures, caused by expression of EHD3 double mutant, a visible physiological effect on transferrin recycling could be detected. These results are in accord with findings showing that knock down of EHD3 caused a delay in transferrin recycling [21]. Based on the above, we conclude that EHD3 SUMOylation is involved in the formation of tubular ERC and therefore, affects both EHD3 and EHD1 (Fig 6) localization to the peripheral tubular recycling endosomes and that this SUMOylation-induced localization to recycling endosomal tubules has an important role in recycling.

Since we observed an almost complete loss of EHD3 from ERC tubules due to the elimination of its SUMOylation, a key question is whether SUMOylation of EHD3 is important for EHD3 localization to the ERC tubules or this modification actually induces ERC tubulation itself. Membrane tubulation plays an important role in intracellular trafficking between different endosomal compartments [43, 44], since it enables efficient movement of cargo [44–46]. Previous studies demonstrated that inhibition of membrane tubulation in the endocytic pathway results in a delay in transferrin and transferrin receptor recycling [44, 47]. In a recent work, Cai et al. suggested that EHD3 tubulates endosomal membranes [19]. *In-vitro* EHD-mediated tubulation [16] has been shown to occur in two steps: 1. Dimerization of an EHD protein and membrane binding via ionic interactions, and 2. Oligomerization around the lipids, in a ring like shape, which leads to membrane elongation. Since our results showed that SUMOylation does not control EHD3 dimerization (Fig 5A and 5B), it seems as an essential factor in its oligomerization.

The impact of SUMOylation on oligomerization has already been documented for other proteins. Thus, SUMOylation-modulated oligomerization of the endocytic protein dynamin [48], which shares high similarity with EHDs in their nucleotide binding domain (dynamin binds GTP while EHDs bind ATP) [16, 18] reviewed by: [12]. SUMOylation of dynamin inhibits its oligomerization and downregulates dynamin-mediated endocytosis of transferrin [48]. Thus, while SUMOylation of dynamin regulates its disassembly from the membrane, SUMOylation of EHD3 seems to mediate its oligomerization and membrane tubulation.

SUMOylation may affect endocytosis of proteins. The two kainate receptor subunits, GluR6 and GluK2, were reported to undergo SUMOylation. GluR6 exhibited an elevated level of SUMOylation upon kainate treatment. Reduced GluR6 SUMOylation caused an inhibition of kainate receptor endocytosis [49]. On the other hand, SUMOylation of GluK2 promoted kainite receptor endocytosis [50, 51]. Arrestins are well-established regulators of G protein-coupled receptor (GPCR) desensitization, trafficking, and signaling. Arrestin-3 undergoes SUMO1 dependent SUMOylation upon activation of β2-adrenergic receptor (β2AR). Depletion of Ubc9 enzyme or expression of SUMO-deficient arrestin-3 mutant blocked β2AR internalization, suggesting that SUMOylation of arrestin-3 mediates GPCR endocytosis [52].

In general, SUMOylation involves changes in the target proteins that either make them susceptible to modifications or regulate their activity and/or localization [53]. Thus, SUMOylated SNF1, the yeast ortholog of the AMP-activated protein kinase (AMPK) undergoes ubiquitination upon SUMOylation and degradation by the ubiquitin-proteasome pathway [54]. RanGAP1 serves as a guanine activating protein (GAP) for RanGAP, and circulates between the cytoplasm and the nucleus. SUMOylation of RanGAP1 modulates its localization. More specifically, SUMO modification of this protein enhances its interaction with nuclear pore complex protein RanBP2 and subsequently contributes to its entry to the nucleus [31, 55].

SUMOylation of proteins is also involved in their exit from the nucleus to the cytoplasm. One example is Smad3, a known signal transducer in TGFβ signaling cascade, which shuttles to the nucleus due to TGFβ stimulation. Its exit from the nucleus depends on its SUMOylation [56]. Recently, we found that EHD2 shuttles to the nucleus, where it serves as a co-repressor of transcription and the exit of EHD2 from the nucleus depends on its SUMOylation [30]. This was also shown for the *Arabidopsis* homolog, AtEHD2 [57].

Our results, showing that SUMOylation of EHD3 is essential for maintaining proper recycling, indicate the delicate balance required in cell trafficking. Changes in the levels of SUMOylated protein may alter the kinetics of this progress. In this context, the cell may use SUMOylation and other post translational modifications in order to fine tune the levels of endocytosis and trafficking, and adjust them to the outer environment.

To summarize, in the present work we found that SUMOylation of EHD3 is crucial for its localization to the tubular ERC structures and proper recycling of transferrin from the ERC back to the cell surface, implying that SUMOylation of EHD3 is important for the integrity of tubular ERC structures and most likely for their formation.

## Supporting Information

S1 Fig EHD3 undergoes SUMOylation. **A.** COS cells were transiently transfected with wt GFP-EHD3 (WT) or its SUMOylation mutants: GFP-EHD3K315A (K3115A), GFP-EHD3K511A (K511A), GFP-EHD3K(315+511)A [K(315+511)A]. Twenty-four hours later cells were fixed with 4% paraformaldehyde and visualized using confocal microscopy. Arrows indicate tubular structures. Scale bars represent 10 μm. **B.** Quantification of signal intensity obtained from tubular structures (%) of either wt or its SUMOylation mutants. ***P<0.0001. Eighty to 100 cells were analyzed for each type of EHD3 variant.
(TIF)

S2 Fig EHD3 undergoes SUMOylation. Original blots of Fig 3A. Analyzed by Bio-Rad ChemiDoc XRS+.
(TIF)

S3 Fig The effect of SUMOylation on dimerization of EHD3. Original blots of Fig 4A, upper panel. Analyzed by Kodak X-OMAT 2000 Processor.
(TIF)

S4 Fig The effect of SUMOylation on dimerization of EHD3. Original blots of Fig 4A, lower panel. Analyzed by Kodak X-OMAT 2000 Processor.
(TIF)

S5 Fig The effect of EHD3 SUMOylation on EHD1 localization. Original blots of Fig 6A. Analyzed by Kodak X-OMAT 2000 Processor.
(TIF)
