# Supplementary figures and images for: SUMOylation of EHD3 Modulates Tubulation of the Endocytic Recycling Compartment
Source: PLoS One. 2015 Jul 30;10(7):e0134053. doi: 10.1371/journal.pone.0134053 (PMC4520680; doi:10.1371/journal.pone.0134053)

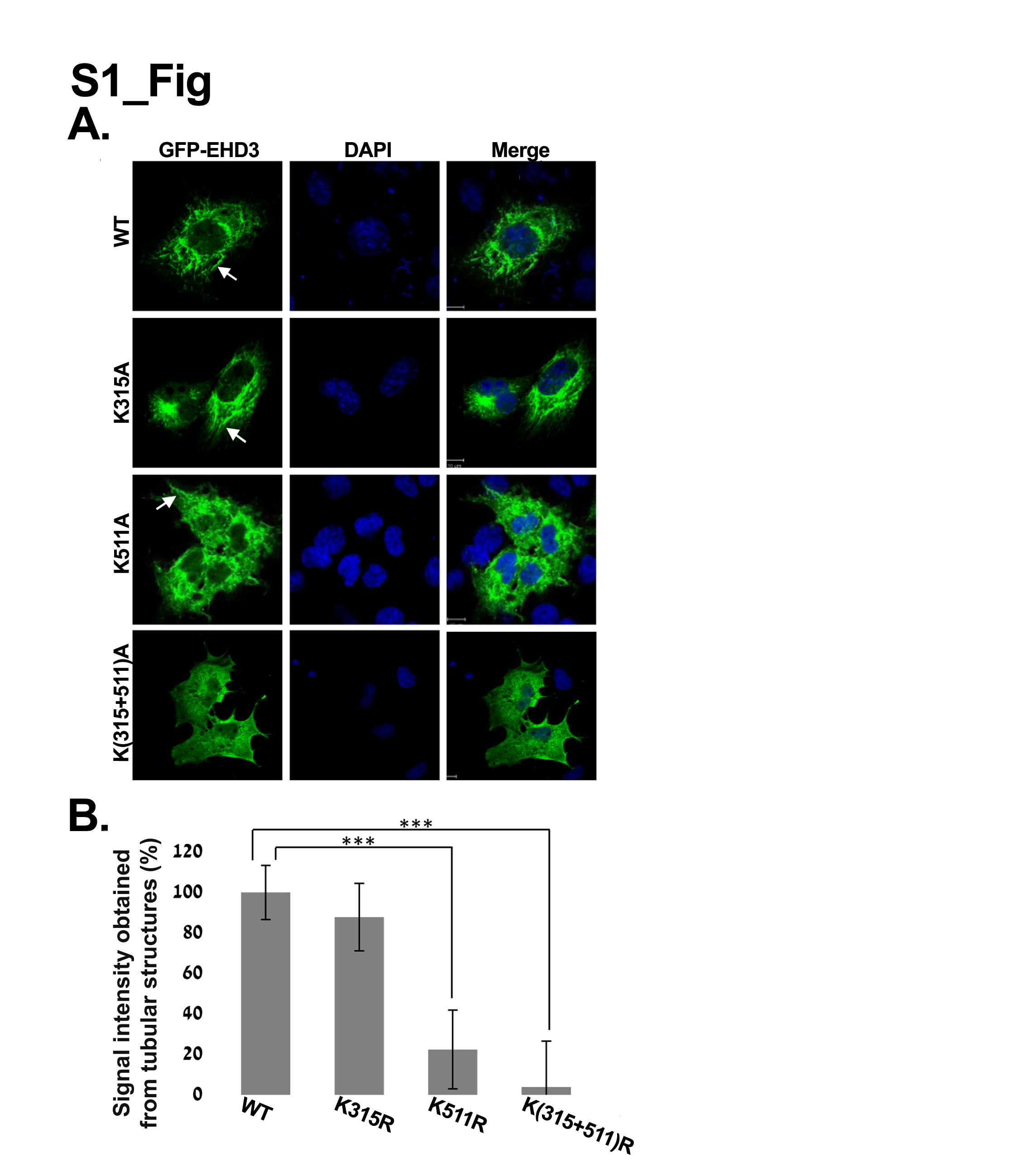

Supplement: S1 Fig — A. COS cells were transiently transfected with wt GFP-EHD3 (WT) or its SUMOylation mutants: GFP-EHD3K315A (K3115A), GFP-EHD3K511A (K511A), GFP-EHD3K(315+511)A [K(315+511)A]. Twenty-four hours later cells were fixed with 4% paraformaldehyde and visualized using confocal microscopy. Arrows indicate tubular structures. Scale bars represent 10 μm. B. Quantification of signal intensity obtained from tubular structures (%) of either wt or its SUMOylation mutants. ***P<0.0001. Eighty to 100 cells were analyzed for each type of EHD3 variant. (TIF) [file pone.0134053.s001.tif]

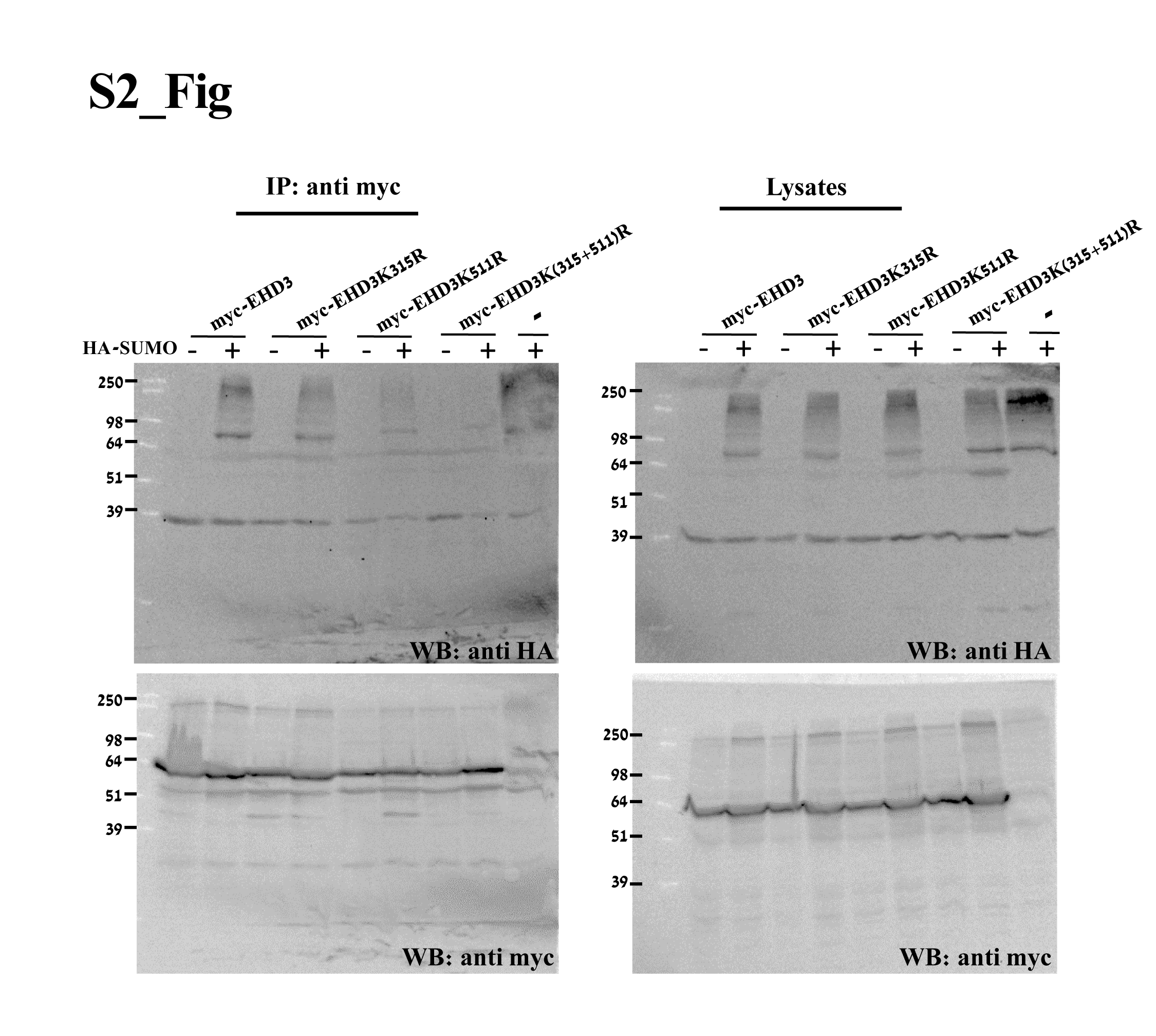

Supplement: S2 Fig — Original blots of Fig 3A. Analyzed by Bio-Rad ChemiDoc XRS+. (TIF) [file pone.0134053.s002.tif]

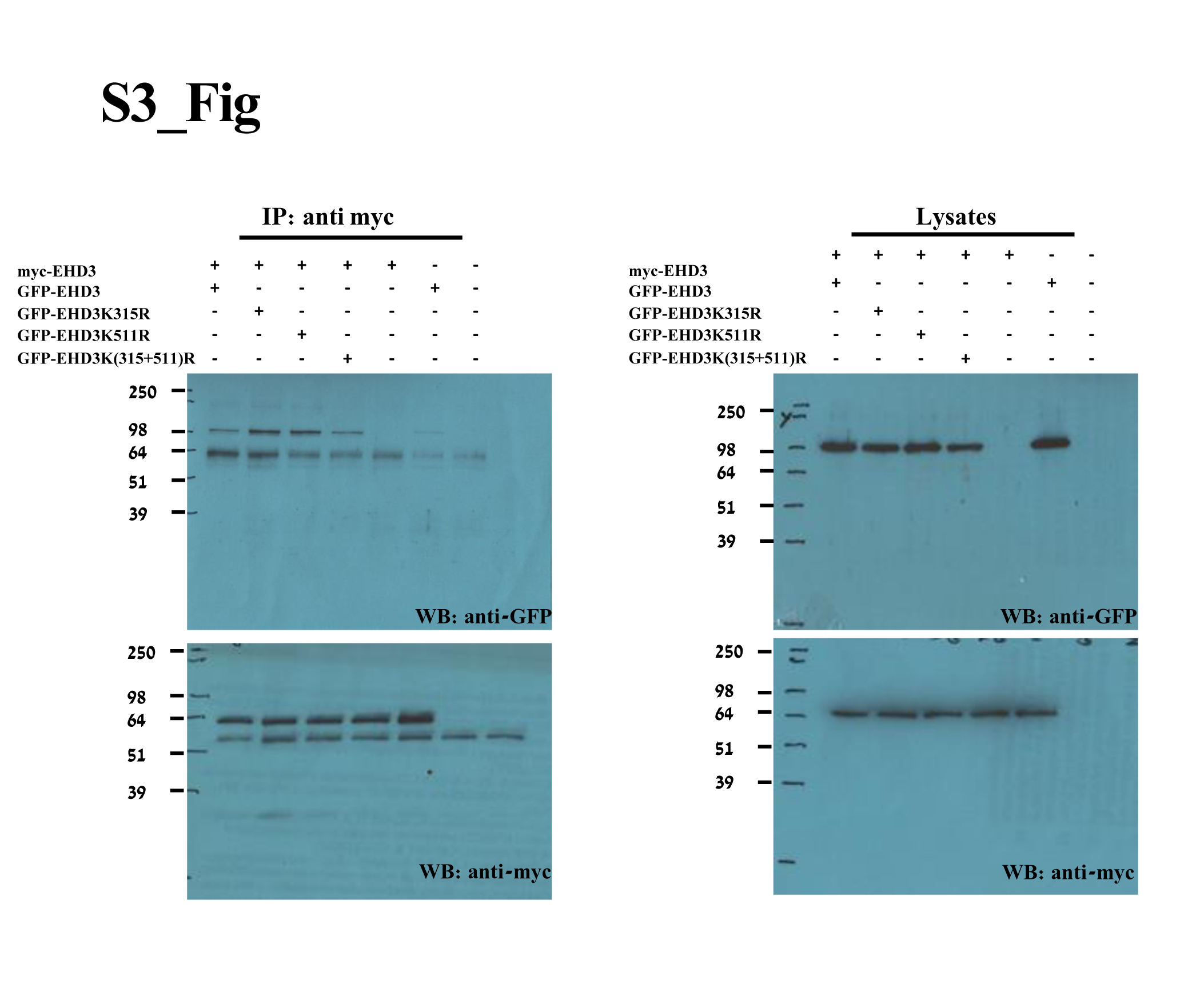

Supplement: S3 Fig — Original blots of Fig 4A, upper panel. Analyzed by Kodak X-OMAT 2000 Processor. (TIF) [file pone.0134053.s003.tif]

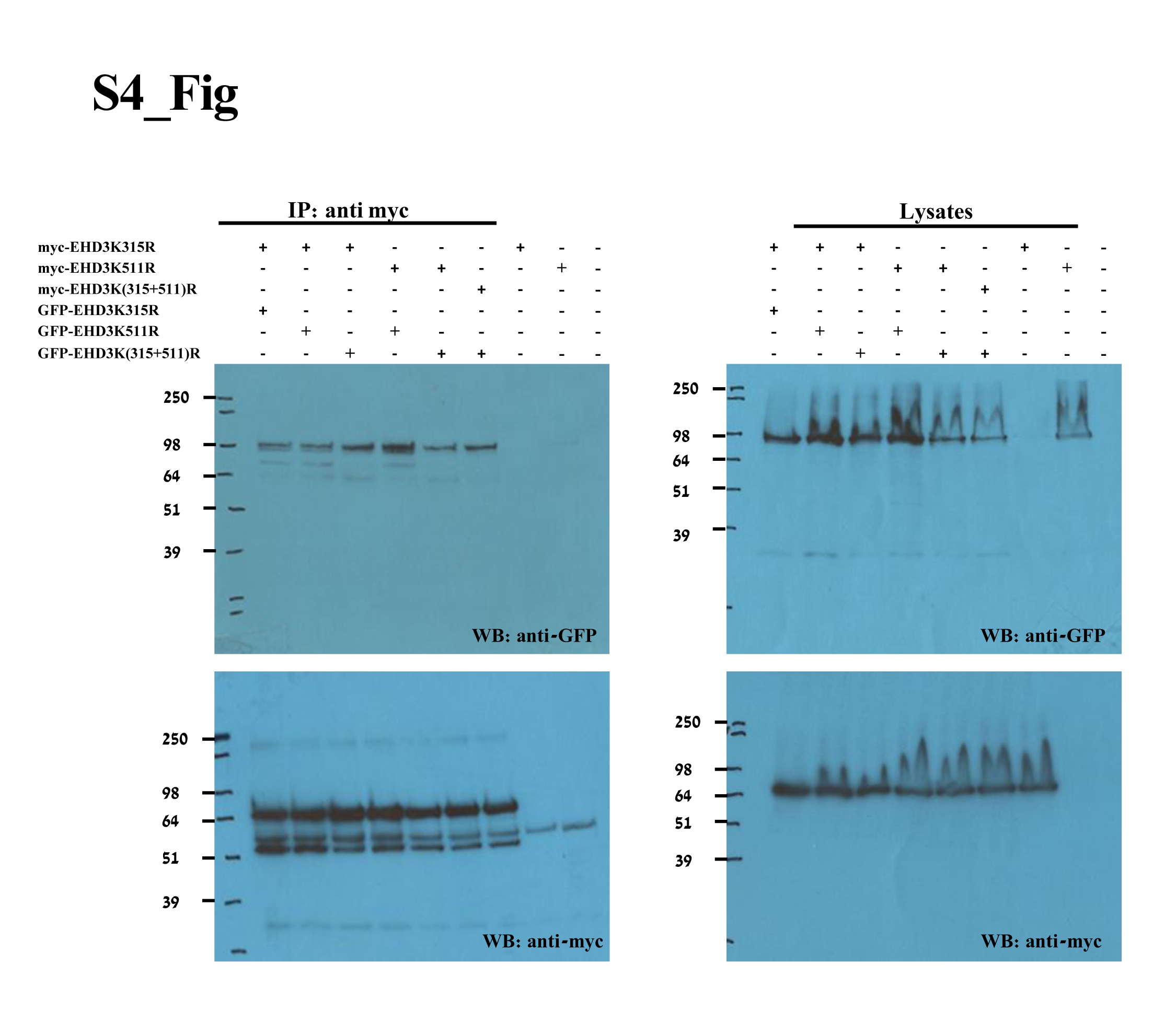

Supplement: S4 Fig — Original blots of Fig 4A, lower panel. Analyzed by Kodak X-OMAT 2000 Processor. (TIF) [file pone.0134053.s004.tif]

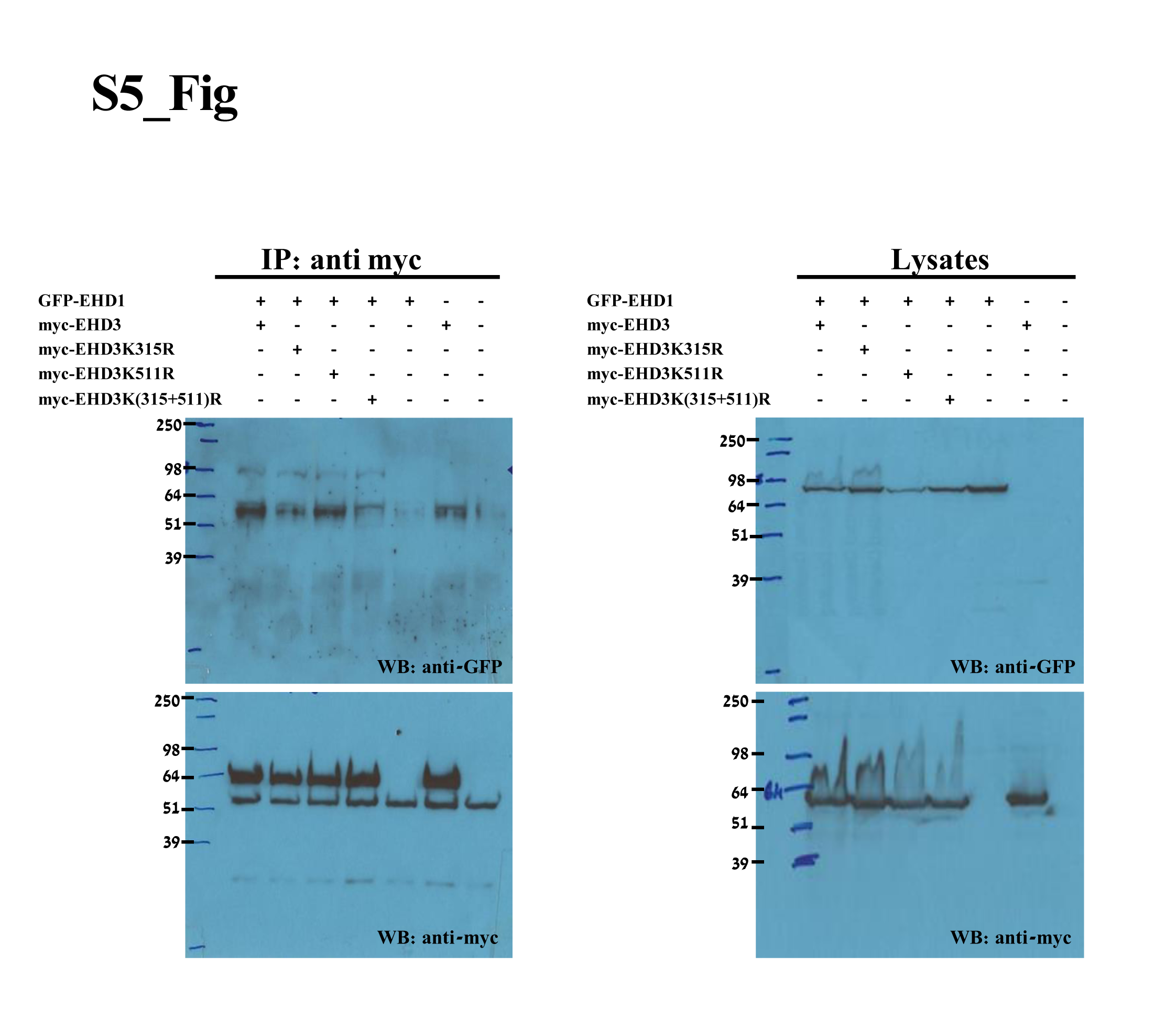

Supplement: S5 Fig — Original blots of Fig 6A. Analyzed by Kodak X-OMAT 2000 Processor. (TIF) [file pone.0134053.s005.tif]
